# A Rare Scrotal Tumor: Epididymal Cavernous Hemangioma

**DOI:** 10.1155/2018/4259563

**Published:** 2018-11-06

**Authors:** Omar Karray, Mohamed Ali Ben Chehida, Ahmed Sellami, Kheireddine Mrad Daly, Zied Mahjoubi, Alia Zehani, Sami Ben Rhouma, Yassine Nouira

**Affiliations:** ^1^Urology Department, La Rabta Hospital, Tunis, Tunisia; ^2^Pathology Department, La Rabta Hospital, Tunis, Tunisia

## Abstract

**Introduction:**

Paratesticular tumors are rarely observed among scrotal neoplasm. Various types of benign lesions are described. Cavernous hemangioma belongs to uncommon epididymal benign tumors. Clinical and sonographic features are not conclusive and diagnosis requires histological confirmation.

**Case Presentation:**

Authors report a case of an epididymal hemangioma in a 56-year-old patient, consulting for a painful scrotal swelling. As malignancy was suspected, he underwent inguinal orchiectomy. Histological examination confirmed the diagnosis of cavernous epididymal hemangioma. Clinical and therapeutic aspects of this rare entity are discussed.

**Conclusion:**

Epididymis is an infrequent location of cavernous hemangioma. Diagnosis is rarely made preoperatively as symptoms and radiological aspects are not specific. Conservative surgery must be attempted once feasible for aesthetic and functional purposes.

## 1. Introduction

Vascular intrascrotal tumors, arising primarily from the epididymis, are not commonly documented in daily practice. The distinction between benign and malignant epididymal tumors is not obvious, as clinical, radiological, and even operative features are not formally evocative.

Herein, authors report a case of a rarely observed epididymal tumor, the cavernous hemangioma. Clinical, sonographic, operative, and histological features will be described and discussed.

## 2. Case Presentation

A fifty-six-year-old patient, father of two, trader, with no past medical history, consulted for a scrotal swelling. History-taking revealed neglected left scrotal pain for three years, increasing progressively. He denied any episodes of orchitis, scrotal traumatism, or micturition disorders. On physical examination, a three-centimeter tender and indurated mass was palpable on the epididymal caput. The right scrotum and the inguinal region's examination was without abnormalities. Routine blood testing and semen analysis were normal. Urinalysis was aseptic. Testicular tumor markers bioassay, including Alpha-fetoprotein, lactate dehydrogenase, and human chorionic gonadotropin, was performed. They were all in the normal rates.

**Figure 1:**
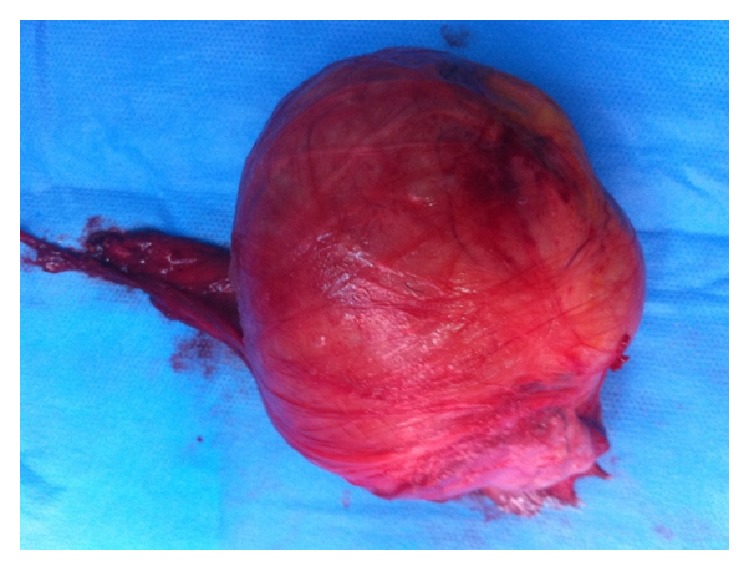
Operative specimen: left orchiectomy, with a 7-centimeter regular solid mass of the epididymis caput.

Scrotal ultrasound showed a heterogeneous tumefaction of the left epididymal caput, measuring 70 millimeters. The epididymal cauda and the left testis had a normal aspect. As malignancy was strongly suspected, surgery was performed after sperm cryopreservation. A left scrotal approach was done in order to attempt an epididymectomy. A 70-millimeter regular and solid mass was observed in the epididymal caput. As inflammatory phenomena were intense, epididymal dissection was laborious. It was decided to perform an orchiectomy, sectioning the spermatic cord as near as possible to the external inguinal ring (Figure 1). Postoperative course was uneventful. The patient left the hospital in the first postoperative day.

**Figure 2:**
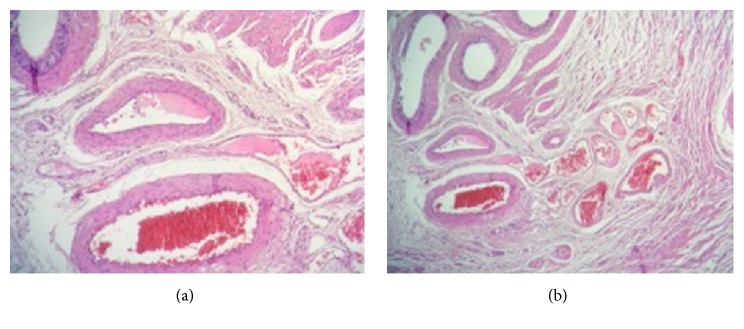
Important vascular proliferation. Vessels are enlarged, laying in an abundant connective tissue.

**Figure 3:**
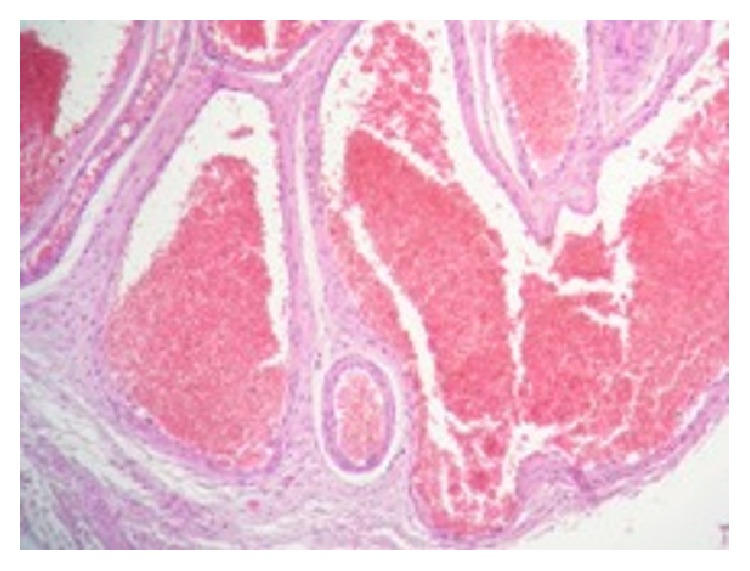
Regular flat endothelial cells bordering the vessels lumen.

Histological examination concerned a 12*∗*7*∗*6-centimeter orchiectomy specimen. Testicular parenchyma had a normal microscopic aspect. The epididymis caput contained a regular hemorrhagic nodule measuring 55 millimeters. Microscopic examination of the nodule revealed a vascular proliferation made of enlarged vessels, sometimes cystic, separated by an abundant connective tissue (Figures 2(a) and 2(b)). Vascular cavities were congestive and bordered with a single layer of regular endothelial flat cells (Figure 3). The vessel's wall was thickened, with variable degrees of adventitial fibrosis. The lesions were concordant with the diagnosis of an epididymal cavernous hemangioma.

The patient was examined three months and afterwards annually for three years. Scrotal examination and ultrasound did not reveal any signs of local recurrence. The patient did not complain of any erectile or sexual dysfunction.

## 3. Discussion

Paratesticular tumors are heterogeneous and infrequent among scrotal neoplasms. They concern the spermatic cord in 90% of the cases, and less frequently the epididymis and the testicular tunics. Malignancy is observed in almost 30% of the cases [1]. Epididymal benign tumors are essentially divided into three types: adenomatoid tumors, leiomyomas, and epididymal cystadenomas [2].

Hemangioma is defined as a benign mesenchymal tumor. Different types are described. They may be capillary, cavernous, veinous, and arteriovenous. These subtypes may be observed in the same tumor [3].

Epididymis represents only 1% of all hemangiomas, which concern mainly the liver, the spleen, and the musculoskeletal system [4]. The first case of epididymal hemangioma was reported by Rosenthal in 1946. It can be associated with testicular hemangioma, as described by Robertson et al. [5].

Clinical presentation is not specific. The tumor may occur at any age. The main complaint is usually a scrotal swelling. Tenderness is often reported. It is difficult to distinguish benign and malignant tumors based only on the physical examination. Spermatogenesis disorders are reported and can be explained by the increase of scrotal temperature by the hemangioma [6].

Scrotal ultrasonography is rarely conclusive. Benign tumors usually present as homogeneous and hyperechoic masses. Hemangioma may be hypo- or hyperechoic. A heterogeneous aspect has also been described [7]. Blood flow may be missing in Doppler evaluation. Even if observed, germ cell tumors, like choriocarcinoma, are therefore suspected [6].

Magnetic resonance imaging can be rewarding in describing the tumor, its location, and its extension to the other paratesticular elements [8].

Definitive diagnosis is histological. Macroscopically, the epididymis is enlarged and contains blood clots on section. Microscopic examination shows epididymal tubules separated by blood-filled ectatic vascular spaces, without atypia or malignancy lesions [9].

As malignancy cannot be ruled out on clinical or radiological features, surgery is mandatory.

In most of the cases, like in the one we report, orchiectomy was performed. That being said, a conservative surgery is recommended once feasible. It depends on the preoperative surgeon evaluation of the lesions and the frozen section examination. A complete epididymectomy allows conserving the testis and avoids recurrence [4]. In our case, epididymectomy was attempted. As the epididymis was inflammatory and adherent to the testis, dissection and liberation of the epididymis was difficult. Thus, orchiectomy was decided.

Prognosis is favorable and recurrence is essentially related to incomplete epididymal excision [4]. Malignant degeneration is not described [9]. Follow-up concerns mainly the remaining testis if orchiectomy was realized. Psychological and aesthetic aspects must be considered and a testicular prosthesis should be proposed postoperatively.

## 4. Conclusion

A better knowledge of benign paratesticular tumor allows realizing conservative surgery, with a favorable outcome and an acceptable aesthetic, psychological, and sexual impact. A thorough preoperative evaluation and eventually frozen section examination allow avoiding an unnecessary orchiectomy.

## Data Availability

The authors will make readily reproducible and freely available materials described in this report to any scientist wishing to use them, without breaching the patient confidentiality.
